# Novel concept to guide systolic heart failure medication by repeated biomarker testing—results from TIME-CHF in context of predictive, preventive, and personalized medicine

**DOI:** 10.1007/s13167-018-0137-7

**Published:** 2018-05-13

**Authors:** Nasser Davarzani, Sandra Sanders-van Wijk, Micha T. Maeder, Peter Rickenbacher, Evgueni Smirnov, Joël Karel, Thomas Suter, Rudolf A. de Boer, Dirk Block, Vinzent Rolny, Christian Zaugg, Matthias E. Pfisterer, Ralf Peeters, Hans-Peter Brunner-La Rocca, for the TIME-CHF investigators

**Affiliations:** 1 Department of Data Science and Knowledge Engineering, Maastricht University, St. Servaasklooster 39, P.O. Box 616, 6200 MD Maastricht, the Netherlands; 2 Department of Cardiology, Maastricht University Medical Center, Maastricht, the Netherlands; 3 GROW School for Oncology and Developmental Biology, Department of Pathology, Maastricht University Medical Center, Maastricht, the Netherlands; 4 Department of Cardiology, Kantonsspital St. Gallen, St. Gallen, Switzerland; 5 Division of Cardiology, University Hospital Bruderholz, Bruderholz, Switzerland; 6 Department of Cardiology, University Hospital Berne, Berne, Switzerland; 7 Department of Cardiology, University Medical Center Groningen, Groningen, the Netherlands; 8 Roche Diagnostics GmbH, Penzberg, Germany; 9 Roche Diagnostics International, Rotkreuz, Switzerland; 10 Department of Cardiology, University Hospital Basel, Basel, Switzerland

**Keywords:** Heart failure, Biomarker, Heart failure medication, Predictive preventive personalized medicine, Generalized estimating equations

## Abstract

**Background:**

It is uncertain whether repeated measurements of a multi-target biomarker panel may help to personalize medical heart failure (HF) therapy to improve outcome in chronic HF.

**Methods:**

This analysis included 499 patients from the Trial of Intensified versus standard Medical therapy in Elderly patients with Congestive Heart Failure (TIME-CHF), aged ≥ 60 years, LVEF ≤ 45%, and NYHA ≥ II, who had repeated clinical visits within 19 months follow-up. The interaction between repeated measurements of biomarkers and treatment effects of loop diuretics, spironolactone, β-blockers, and renin-angiotensin system (RAS) inhibitors on risk of HF hospitalization or death was investigated in a hypothesis-generating analysis. Generalized estimating equation (GEE) models were used to account for the correlation between recurrences of events in a patient.

**Results:**

One hundred patients (20%) had just one event (HF hospitalization or death) and 87 (17.4%) had at least two events. Loop diuretic up-titration had a beneficial effect for patients with high interleukin-6 (IL6) or high high-sensitivity C-reactive protein (hsCRP) (interaction, *P* = 0.013 and *P* = 0.001), whereas the opposite was the case with low hsCRP (interaction, *P* = 0.013). Higher dosage of loop diuretics was associated with poor outcome in patients with high blood urea nitrogen (BUN) or prealbumin (interaction, *P* = 0.006 and *P* = 0.001), but not in those with low levels of these biomarkers. Spironolactone up-titration was associated with lower risk of HF hospitalization or death in patients with high cystatin C (CysC) (interaction, *P* = 0.021). β-Blockers up-titration might have a beneficial effect in patients with low soluble fms-like tyrosine kinase-1 (sFlt) (interaction, *P* = 0.021). No treatment biomarker interactions were found for RAS inhibition.

**Conclusion:**

The data of this post hoc analysis suggest that decision-making using repeated biomarker measurements may be very promising in bringing treatment of heart failure to a new level in the context of predictive, preventive, and personalized medicine. Clearly, prospective testing is needed before this novel concept can be adopted.

**Clinical trial registration:**

isrctn.org, identifier: ISRCTN43596477

**Electronic supplementary material:**

The online version of this article (10.1007/s13167-018-0137-7) contains supplementary material, which is available to authorized users.

## Introduction

Medical therapy for chronic heart failure (HF) with reduced ejection fraction (HFrEF) has evolved greatly over the past decades [1]. Several medication classes including renin-angiotensin system (RAS) inhibitors encompassing angiotensin-converting enzyme (ACE) inhibitors and angiotensin-receptor blockers (ARBs), β-blockers, and mineralocorticoid receptor antagonists (MRAs) have been shown to improve prognosis in HFrEF and are therefore recommended in high doses by current guidelines [2, 3]. In addition, (loop) diuretics are recommended for decongestion to relieve symptoms [2, 3]. However, in clinical practice, it is often difficult to implement all medication classes and specially to reach guideline-recommended dosages, especially in elderly and comorbid patients [4].

N-Terminal pro B-type brain natriuretic peptide (NT-proBNP) is a widely used and accepted as diagnostic and prognostic biomarker in HF [5]. The level of NT-proBNP is changing as response to therapy [6, 7]. Therefore, it was suggested as a tool to tailor and intensify medical HF therapy. Several trials and meta-analyses suggest that performing repeated measurements of (NT-pro)BNP may help to establish guideline-recommended medical therapy in HFrEF patients to improve outcome [8, 9]. However, the large GUIDE-IT trial [10] that aimed to prove this concept in a sufficiently large patient population was stopped early due to futility (https://dcri.org/dcri-announces-halt-guide-trial/). Moreover, guiding HF therapy by a single marker is limited because one biomarker cannot cover the extensive pathophysiological pathways involved in HF. In fact, the approach to guide therapy using (NT-pro)BNP is based on the idea that patients at highest risk of poor outcome are in need of intensified therapy, but there is no specific tailoring of individual drugs. However, HF is a very complex disease requiring an integrated approach [11] and different medication classes in HF interfere with different pathways. Therefore, a combination of biomarkers that reflect these pathways may be better suited to indicate which medication class is most important to up- or possibly down-titrate in a specific patient. That may lead towards predictive, preventive, and personalized medicine in HF, based on an integrated approach as suggested recently [12]. Such an attempt has, however, never been made. As a first step towards the development of a biomarker-guided treatment algorithm for personalized medical HFrEF therapy, we, therefore, investigated the interaction between multiple repeatedly measured biomarkers and the response to the four most important classes of HF medication regarding the risk of HF hospitalization or death. Thus, the main objective of the study, as a purely hypothesis-generating study, was to explore which biomarkers in repeated testing would be most predictive of the response to HF drugs during follow-up.

## Methods

### Study and design

Since an important prerequisite to address the objective was the availability of detailed data on patient characteristics at different time points, medication over time, and repeatedly measured multiple biomarkers, we used the database of the Trial of Intensified versus standard Medical therapy in Elderly patients with Congestive Heart Failure (TIME-CHF) for this analysis. The study design, results, and methods of the TIME-CHF have been previously published in detail [13, 14]. In brief, the study included 499 patients aged 60 years or older with symptomatic HF (NYHA ≥ II), left ventricular ejection fraction (LVEF) ≤ 45%, a history of HF hospitalization within the preceding year, and a NT-proBNP level higher than twice the upper limit of normal, from 15 centers in Switzerland and Germany. Some exclusion criteria applied (e.g., valvular heart disease needing surgery, recent acute coronary syndrome percutaneous coronary intervention or coronary artery bypass graft surgery, serum creatinine ≥ 220 μmol/l, for the details see [13]), but on average, the patients did not differ much from those included in large registries. Patients were randomized to either standard (symptom-guided) or intensified (NT-BNP-guided) medical therapy.

Patients visits took place at the baseline (zero month), were followed up for 1, 3, 6, 12, and 18 months. For each patient, time to recurrence of clinical events was recorded, up to 5½ years. The primary endpoint for the present analysis was the combined endpoint of HF hospitalization or death during the 18-month trial period plus 1 month of additional follow-up, i.e., 19 months in total.

History was taken, patients were clinically investigated, and blood samples were drawn at every visit. Samples were stored at − 80 °C until analysis. At the end of the trial, 20 biomarkers were measured from these stored samples from all available visits. Selection of biomarkers was based on the representation of different pathways that are known to reflect important pathophysiological pathways as previously reported [15]. Daily medication doses for all drugs including the four drug classes investigated in this analysis, i.e., β-blockers, RAS inhibitors, spironolactone, and loop diuretics, were available as described [16].

### Data description

The study contains three types of covariates as presented in Table 1.

**Table 1:** Baseline characteristics and biomarkers and the average drug dosages at the first month in patients without versus with event (HF hospitalizations or death) within 19 months

Variables	All patients (*n* = 499)	No event (*n* = 312)	One or more events (*n* = 187)	*P* value*
Baseline characteristics				
Age (years), mean (sd)	76.1 (7.5)	75.1 (7.5)	77.9 (7.2)	0.000
Male gender (%)	327 (65.5)	200 (64.1)	127 (67.9)	0.386
CAD (%)	287 (57.5)	153 (49)	134 (71.7)	0.000
Charlson score, median [IQR]	3 [2–4]	3 [2–4]	3 [2–5]	0.000
LVEF, mean (sd)	29.8 (7.8)	29.7 (7.7)	29.9 (8.0)	0.844
Kidney_disease (%)	277 (55.5)	150 (48.1)	127 (67.9)	0.000
BPsyst, mean (sd)	118.5 (18)	119.6 (18)	116.7 (18.1)	0.098
Rales (%)	209 (42.1)	100 (35.5)	99 (52.9)	0.000
NYHA > II (%)	371 (74.3)	219 (70.2)	152 (81.3)	0.006
Biomarkers, median [IQR]				
sFlt	98.8 [81–128]	93.9 [77.3–124.2]	105.6 [88.4–132.8]	0.000
GDF15	3940 [2697–5891]	3530 [2416–5125]	4786 [3433–7183]	0.000
CysC	1.7 [1.4–2.1]	1.6 [1.3–1.9]	1.9 [1.6–2.4]	0.000
Ferritin	152 [80–258]	159 [85–261]	151 [66–248]	0.314
IL6	7.3 [3.9–14.1]	6.6 [3.5–11.9]	9.3 [4.6–16.8]	0.001
PLGF	22.6 [18.3–26.5]	22.3 [18.0–26.1]	22.8 [18.8–27.1]	0.222
SHBG	30.1 [22.3–40.6]	30.1 [22.7–42.3]	30.1 [21.4–38.9]	0.232
sTFR	4.1 [3.2–5.4]	3.9 [3.0–5.2]	4.3 [3.3–5.9]	0.016
hsTnT	33.6 [19.1–62.7]	28.6 [17.9–53.3]	45.8 [24.4–85.4]	0.000
tP1NP	36.7 [23.7–55.5]	34.9 [23.7–51.5]	38.2 [23.7–62]	0.170
Uric	7.7 [6.1–9.2]	7.3 [5.9–8.8]	8 [6.7–9.5]	0.003
BUN	10.4 [7.6–13.5]	9.4 [7.3–12]	12.5 [8.6–16.1]	0.000
sST2	35.9 [26–54]	32.5 [24–45.5]	43.5 [31–64.2]	0.000
NT-proBNP	4194 [2270–7414]	3675 [1831–6301]	5465 [3049–9743]	0.000
Creatinine	109 [88–141]	102 [84–127]	132 [99–157]	0.000
hsCRP	6.7 [2.5–15.8]	5.5 [1.9–14.8]	8.9 [3.6–20.4]	0.008
PREA	0.19 [0.15–0.23]	2.00 [0.15–0.24]	0.17 [0.14–0.22]	0.015
OPN	26.0 [17.0–40.9]	22.6 [16.1–33.6]	33.5 [21.2–55.2]	0.000
Mimican	116 [85.9–164]	107 [84.1–145]	143 [93.4–198]	0.000
IGFBP7	242 [201–291]	226 [197–274]	265 [221–315]	0.000
Medications, median [IQR]				
RAS inhibitors	59.7 [44.3–100]	59.6 [44.3–100]	50 [40.3–100]	0.048
β-Blockers	25 [11.7–50]	25 [12.1–50]	25 [10.5–46.1]	0.119
Loop diuretics	60.6 [40–92.4]	43.2 [33.1–80]	80 [40–129]	0.000
Spironolactone	1.6 [0–25]	0 [0–25]	12.5 [0–25]	0.003

*P value of testing whether the variables are the same in the mean (for continuous normally distributed variables) or median (for continuous non-normally distributed variables) or percentage (for categorical variables) between those patients not hospitalized and those hospitalized or died (two-sided *t* test or Mann-Whitney *U* test for continuous variables and χ^2^ test for categorical variables)

*IQR* interquartile range

#### Patient characteristics

Age, gender, coronary artery disease (CAD), Charlson comorbidity score, LVEF, and history of kidney disease were recorded only at the baseline visit. Systolic blood pressure (BPsyst) and rales on auscultation were recorded at every visit. These eight characteristics were used as covariates in the multivariable model in the present analysis.

#### Biomarkers

Based on the pathophysiological pathways considered to play an important role in heart failure and previously findings on the prognostic significance [15], the following 20 biomarkers were measured at every visit: soluble fms-like tyrosine kinase-1 (sFlt), growth differentiation factor 15 (GDF-15), cystatin-c (CysC), ferritin, interleukin-6 (IL6), placental-like growth factor (PLGF), sex hormone-binding globulin (SHBG), soluble transferrin receptor (sTFR), high-sensitivity troponin T (hsTnT), type 1 procollagen N-terminal pro B-type peptide (tP1NP), uric acid (uric), blood urea nitrogen (BUN), soluble ST2 (sST2), N-terminal brain natriuretic peptide (NT-proBNP), creatinine, high-sensitivity C-reactive protein (hsCRP), prealbumin (PREA), osteopontin (OPN), mimican, and insulin-like growth factor-binding protein 7 (IGFBP7). The assays used to measure these markers are summarized in the Supplementary Table 1.

#### HF medications

The four most important classes of HF medications were considered for this analysis, i.e., β-blockers, RAS inhibitors, spironolactone, and loop diuretics. Doses of β-blockers and RAS inhibitors were expressed as percentage of target dose as previously reported [17] (e.g., 5 mg of ramipril per day is 50% of the target dose of 10 mg/day). For combination of ACE inhibition and ARB, the relative doses were added and expressed as a combined RAS-inhibitor dose. Spironolactone is given in milligrams as it was the only MRA used in TIME-CHF. Loop diuretics are expressed as equivalent dose of furosemide (i.e., 40 mg of furosemide = 10 mg of torasemide = 1 mg of bumetanide). Use and dose of medication were recorded daily in each patient.

#### Outcome measurements

For the present analysis, any HF hospitalization or death occurring at each month during the 19 months follow-up was considered as outcome events (primary endpoint).

### Statistical methods

Patient characteristics, biomarkers at baseline, and average medication dosages are presented as mean and standard deviation (SD) for continuous normally distributed variables, median and interquartile range for non-normally distributed continuous variables, or as numbers and percentages for categorical variables (Table 1). Variables were compared between those patients without an event and those who experienced an event (i.e., HF hospitalization or death) within 19 months follow-up. Differences in these variables per number of events (none vs. at least one) were assessed using a *t* test for continuous normally distributed variables, a Mann-Whitney *U* test for non-normally distributed continuous variables, and a *χ*^2^ test for categorical variables. All tests were two-sided at a 5% level of significance. Calculations were performed with the use of the SPSS statistical package version 22.0.

In order to explore which biomarkers would be most predictive of the response to HF drugs during follow-up period, we tested whether there was a significant interaction between biomarkers and further treatment effects of the four medication classes in our cohort of HF patients, applying the weighted logistic generalized estimating equations (logistic-GEE) model [18–21]. Logistic-GEE models were performed using R (version 3.3.2, package ‘geepack’).

To this end, we defined a binary outcome variable with a value of one if a given patient was hospitalized for HF or died during a certain time interval of follow-up; otherwise, the value was zero. Note that the outcome values for a given patient can change from one time interval to another and that these outcome values are likely to be correlated. For this analysis, we discretized using time intervals of 1 month and gave more weight to the outcome death (two times of HF hospitalization) when applying the weighted logistic-GEE model [21]. Giving weight of three to the outcome death model resulted to the same findings as weight of two. For patients who either died or withdrew from the study before 19 months, the number of outcome values equals the number of follow-up months. In order to apply the weighted logistic-GEE model, we also included the covariate values at the same time resolution as the outcomes. Therefore, we up-sampled or down-sampled the covariate values to monthly values as follows.

The medication covariates were down-sampled to monthly values by taking the average drug dosage during the previous month. As the first observation of drug dosage, in the absence of a previous month, the dosage at baseline was used. The biomarkers, systolic blood pressure, and rales have been measured at the scheduled follow-up visits (baseline, 1st, 3rd, 6th, 12th, and 18th months); obviously for these six measurements, the covariates take the exact value. Then to get the monthly values between these six visits, we used the last observation carried forward method (LOCF) and put the value of the previous visit. For other fixed baseline characteristics (e.g., presence of coronary artery disease), we used the baseline value at every time interval. The data layout and method layout are illustrated in Supplementary Table 2 and Supplementary Fig. 1, respectively.

In order to test the interaction between a given biomarker and medication in the presence of other patient characteristics, we use the following weighted logistic-GEE model:

$$ {\displaystyle \begin{array}{l}\mathrm{logit}\left({P}_{\mathrm{it}}\right)={b}_0+{b}_1{\mathrm{Age}}_{\mathrm{it}}+{b}_2{\mathrm{Gender}}_{\mathrm{it}}+{b}_3{\mathrm{CAD}}_{\mathrm{it}}+{b}_4{\mathrm{CharlsonScore}}_{\mathrm{it}}+{b}_5{\mathrm{LVEF}}_{\mathrm{it}}+{b}_6{\mathrm{Kidneydisease}}_{\mathrm{it}}\\ {}+{b}_7{\mathrm{Rales}}_{\mathrm{it}}+{b}_8{\mathrm{BPsyst}}_{\mathrm{it}}+{b}_9{\mathrm{Biomarker}}_{\mathrm{it}}+{b}_{10}{\mathrm{Medication}}_{\mathrm{it}}\\ {}+\beta {\mathrm{Biomarker}}_{\mathrm{it}}\times {\mathrm{Medication}}_{\mathrm{it}},\mathrm{i}=1,\dots, 499,\mathrm{t}=0,\dots, 18,\end{array}} $$

where P_it_ is the probability of hospitalization or death in the month following month *t* (*t = 0*, means baseline) for patient *i* and Covariate_it_ is the value of the covariate at month *t* for patient *i*. Biomarker × medication indicates the interaction term and *β* is the interaction coefficient.

In order to investigate whether medications have a different effect on the risk of HF hospitalization or death for certain levels of the biomarkers, the interaction coefficient *β* was tested for all possible paired combinations of medication classes and biomarkers. In this study, we used 1-month time interval for discretizing the follow-up period when using logistic-GEE model (Supplementary Table 2). Therefore, the model analyzed the average effects of the covariates over all time intervals on the outcome of interest. Thus, the estimated coefficients can be interpreted as the average effects of the covariates on the risk of HF hospitalization or death in 1 month.

We used RAMCD-CV [22] (ranking accuracy for models based on clustered data using one-patient-out cross-validation) to estimate the predictive performance of the above logistic-GEE model. This is due to possibly correlated measurements of the same patient that the standard evaluation criteria (such as the area under the ROC curve (AUC)), which assume independence of measurements, cannot be used here. RAMCD-CV can be used for assessing how the results of the above logistic-GEE model generalize to a future data set. RAMCD-CV ranges from 0.0 to 1.0, such that the value of 1.0 means that a randomly selected positive outcome (HF hospitalization or death) always gets a higher score, by the applied logistic-GEE model, than a randomly selected negative outcome (no HF hospitalization or death). In this case, the logistic-GEE model is perfect in differentiating the positive and negative outcomes. In the opposite scenario, the value of RAMCD-CV equals 0.0, and when the logistic-GEE model is an intercept-only model, it is equal to 0.5. We note that when the patients’ measurements are uncorrelated, RAMCD-CV is equal to the AUC [22].

In this paper, only those logistic-GEE models with a good predictive performance—set at a RAMCD-CV of > 0.7—were predefined as being statistically solid and meaningful (see Supplementary Fig. 1).

## Results

**Fig. 1:**
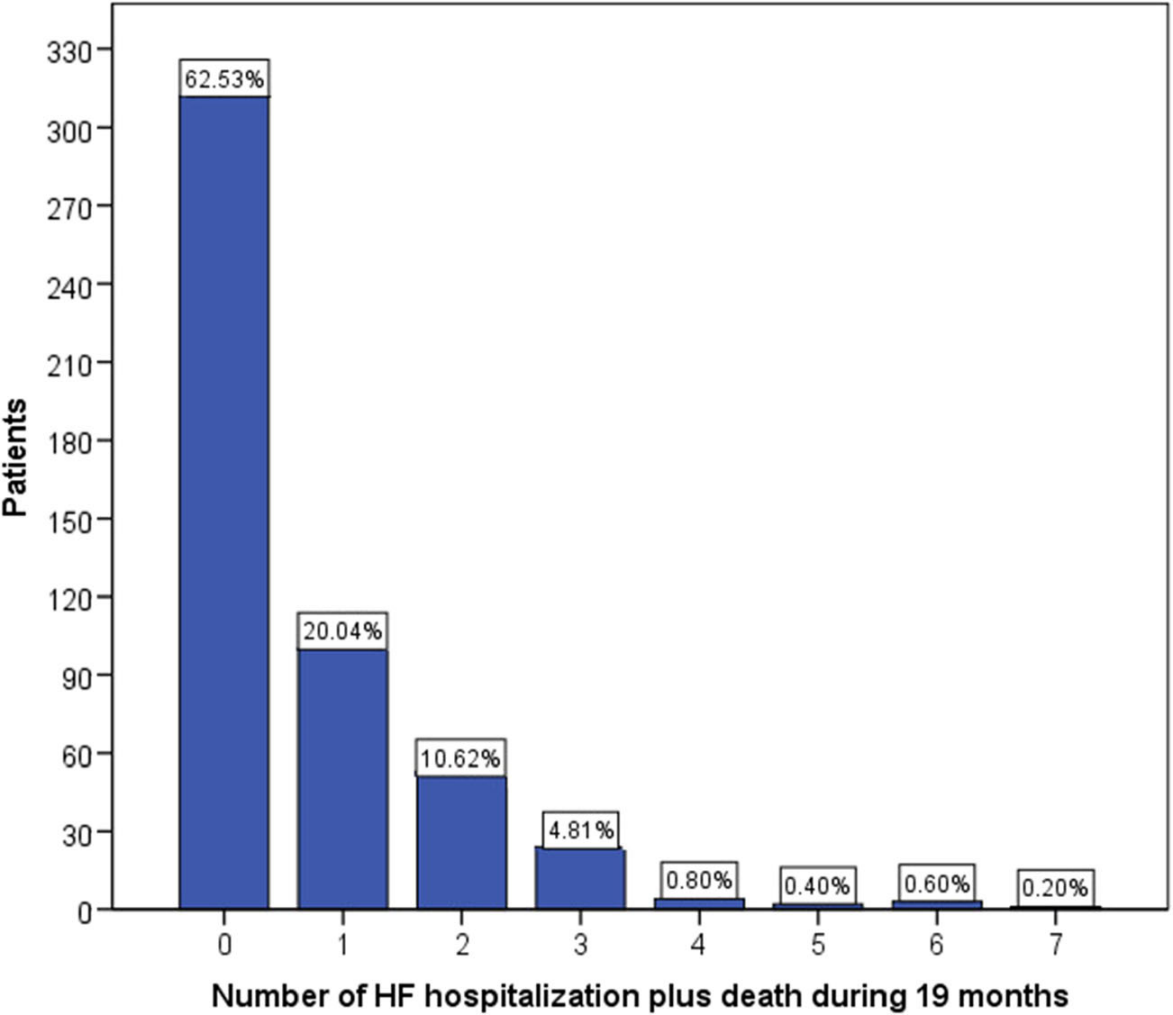
The frequency of HF hospitalizations (including death) during 19-month follow-up

Of the 499 patients, 312 (62.5%) did not experience any HF hospitalization and were alive after 19 months, 100 (20%) had one, and 87 (17.4%) had at least two HF-related events (HF hospitalization or death) within the 19-month follow-up period. The frequency of HF hospitalizations (including death) during the 19-month follow-up is presented in Fig. 1.

Concentrations of biomarkers, baseline characteristics, and average drug dosages during the first month are shown in Table 1. In comparison to patients without event, those with event(s) were older and more likely to have coronary artery disease, kidney disease, rales, more comorbidities, and higher NYHA class. Moreover, they had higher sFlt, GDF15, CysC, IL6, sTFR, hsTnT, uric acid, BUN, sST2, NT-proBNP, creatinine, and hsCRP at baseline and higher average dosages of loop diuretic and spironolactone during the first month, whereas RAS-inhibitor dose was lower.

The *P* values of testing interaction (biomarker × medication) in 84 weighted logistic-GEE models (covering all possible paired combinations of 4 medications and 20 biomarkers and PLGF/sFlt) and their corresponding RAMCD-CVs are presented in Table 2. We note that in our analysis for each medication, we have 21 interaction tests. Therefore, due to the multiple testing, at a 5% level of significance, the Bonferroni correction suggests to reject the null hypothesis (H_0_: *β* = 0) for each test with the *P* value less than 0.05/21 = 0.0024. However, since in this study the results are regarded as hypothesis generating, we consider the interaction tests with *P* value less than 0.05 as being significant. Moreover, *P* value adjustments may raise several practical objections [23]. For example, you may increase the chance of making a type II error [24, 25].

**Table 2:** Results of the interaction (biomarker × medication) coefficient *β* tests using weighted logistic-GEE models (adjusted for age, gender, coronary artery disease as main cause of HF, Charlson Score, left ventricle ejection fraction, kidney disease, rales, systolic blood pressure, medication, and biomarker in logarithmic form) and their corresponding RAMCD-CVs

Biomarkers		β-Blockers	LOOP diuretics	Spironolactone	RAS blockers
sFlt	*β* (CI*)	2.45 (0.37, 4.52)	− 0.8 (2.394,0.793)	2.27 (− 1.83, 6.36)	0.63 (− 1.21, 2.47)
	*P* value**	0.02	0.33	0.28	0.50
	RAMCD-CV***	0.74	0.76	0.73	0.74
GDF15	*β* (CI)	0.33 (− 0.82, 1.49)	0.57 (− 0.902, 2.039)	− 1.10 (− 3.41, 1.22)	− 0.82 (− 1.86, 0.23)
	*P* value	0.57	0.45	0.35	0.13
	RAMCD-CV	0.75	0.76	0.75	0.75
CysC	*β* (CI)	0.21 (− 1.72, 2.14)	0.48 (− 1.16, 2.13)	− 2.73 (− 5.06, − 0.40)	0.66 (− 0.88, 2.19)
	*P* value	0.83	0.57	0.02	0.40
	RAMCD-CV	0.70	0.72	0.70	0.71
Ferritin	*β* (CI)	− 0.58 (− 2.27, 1.11)	0.09 (− 1.52, 1.70)	− 1.49 (− 4.37, 1.40)	− 0.70 (− 2.60, 1.21)
	*P* value	0.50	0.91	0.31	0.47
	RAMCD-CV	0.66	0.70	0.66	0.67
IL6	*β* (CI)	0.76 (− 0.61, 2.12)	− 2.74 (− 4.90, − 0.58)	− 1.22 (− 4.30, 1.86)	− 0.53 (− 1.85, 0.80)
	*P* value	0.27	0.01	0.43	0.43
	RAMCD-CV	0.73	0.75	0.73	0.73
PLGF	*β* (CI)	3.67 (− 1.90, 9.23)	− 1.63 (− 6.60, 3.34)	− 7.90 (− 15.64, − 0.15)	2.01 (− 2.08, 6.09)
	*P* value	0.20	0.52	0.04	0.33
	RAMCD-CV	0.67	0.71	0.66	0.67
SHBG	*β* (CI)	− 0.79 (− 3.64, 2.06)	0.27 (− 2.88, 3.42)	− 2.09 (− 7.75, 3.58)	− 2.70 (− 6.08, 0.68)
	*P* value	0.58	0.86	0.47	0.12
	RAMCD-CV	0.65	0.69	0.65	0.67
sTFR	*β* (CI)	1.35 (− 0.20, 2.90)	− 1.14 (− 2.85, 0.57)	0.19 (− 2.75, 3.12)	− 0.11 (− 1.60, 1.38)
	*P* value	0.09	0.19	0.90	0.88
	RAMCD-CV	0.71	0.73	0.70	0.71
hsTnT	*β* (CI)	− 0.77 (− 2.64, 1.09)	1.11 (− 2.29, 4.51)	1.42 (− 2.20, 5.04)	− 0.07 (− 2.14, 2.00)
	*P* value	0.42	0.52	0.44	0.95
	RAMCD-CV	0.70	0.73	0.70	0.71
tP1NP	*β* (CI)	1.84 (0.04, 3.65)	− 0.66 (− 2.70, 1.38)	− 1.28 (− 5.38, 2.82)	− 0.98 (− 2.79, 0.83)
	*P* value	0.04	0.52	0.54	0.28
	RAMCD-CV	0.67	0.71	0.66	0.67
Uric	*β* (CI)	− 2.92 (− 7.18, 1.35)	1.25 (− 2.23, 4.73)	− 1.98 (− 7.26, 3.31)	3.00 (− 2.29, 8.29)
	*P* value	0.18	0.48	0.46	0.27
	RAMCD-CV	0.67	0.70	0.66	0.68
BUN	*β* (CI)	− 0.17 (− 2.08, 1.73)	2.35 (0.67, 4.03)	− 1.31 (− 3.60, 0.98)	0.54 (− 1.26, 2.34)
	*P* value	0.86	0.00	0.26	0.55
	RAMCD-CV	0.67	0.70	0.67	0.69
sST2	*β* (CI)	0.70 (− 1.47, 2.87)	1.41 (− 1.12, 3.95)	− 0.97 (− 4.91, 2.96)	0.83 (− 1.15, 2.80)
	*P* value	0.53	0.27	0.63	0.41
	RAMCD-CV	0.76	0.77	0.76	0.77
NT-proBNP	*β* (CI)	0.27 (− 1.92, 2.45)	− 0.36 (− 2.84, 2.11)	0.30 (− 3.94, 4.53)	2.25 (− 0.14, 4.63)
	*P* value	0.81	0.77	0.89	0.06
	RAMCD-CV	0.75	0.76	0.74	0.76
Creatinine	*β* (CI)	− 0.25 (− 2.37, 1.87)	1.56 (− 0.12, 3.25)	− 2.60 (− 5.88, 0.69)	0.56 (− 1.42, 2.53)
	*P* value	0.82	0.07	0.12	0.58
	RAMCD-CV	0.69	0.71	0.68	0.70
hsCRP	*β* (CI)	0.98 (− 0.73, 2.68)	− 3.09 (− 4.95, − 1.23)	− 2.20 (− 5.71, 1.31)	0.70 (− 0.94, 2.34)
	*P* value	0.26	0.00	0.21	0.40
	RAMCD-CV	0.72	0.75	0.71	0.72
PREA	*β* (CI)	− 1.02 (− 2.88, 0.85)	3.21 (1.20, 5.23)	− 0.22 (− 3.66, 3.22)	0.17 (− 2.06, 2.41)
	*P* value	0.29	0.00	0.90	0.88
	RAMCD-CV	0.69	0.74	0.69	0.70
OPN	*β* (CI)	1.21 (− 1.07, 3.50)	− 0.07 (− 2.60, 2.46)	− 4.66 (− 10.10, 0.78)	− 0.30 (− 2.51, 1.91)
	*P* value	0.30	0.96	0.09	0.79
	RAMCD-CV	0.70	0.72	0.70	0.71
Mimican	*β* (CI)	0.06 (− 2.25, 2.37)	1.52 (− 1.00, 4.04)	− 3.60 (− 7.48, 0.29)	0.57 (− 1.73, 2.87)
	*P* value	0.96	0.24	0.07	0.63
	RAMCD-CV	0.67	0.70	0.67	0.68
IGFBP7	*β* (CI)	0.52 (− 1.00, 2.03)	− 0.09 (− 1.52, 1.34)	− 3.13 (− 6.98, 0.73)	0.25 (− 1.20, 1.70)
	*P* value	0.50	0.90	0.11	0.74
	RAMCD-CV	0.70	0.73	0.70	0.71
PLGF/sFlt	*β* (CI)	− 2.09 (− 4.8, 0.60)	− 0.16 (− 3.00, 2.67)	− 2.75 (− 7.55, 2.05)	− 0.27 (− 2.68, 2.15)
	*P* value	0.12	0.90	0.26	0.82
	RAMCD-CV	0.71	0.73	0.71	0.72

*RAMCD-CV* ranking accuracy for models based on clustered data using one-patient-out cross-validation

*CI: 95% confidence interval

**P value: *p* value of the interaction effect *β* in weighted logistic-GEE model

***RAMCD-CV for the whole weighted logistic-GEE model including the covariates and the interaction (biomarker × medication)

The results indicate that interactions of (i) sFlt and tP1NP with treatment effect of β-blockers, (ii) IL6, BUN, hsCRP, and PREA with loop diuretics and (iii) CysC and PLGF with spironolactone were significant. That means that these biomarkers might indicate which medication class is most important to up-titrate or possibly down-titrate for improvement of outcome. No significant interactions between biomarkers and RAS inhibitors were found.

**Fig. 2:**
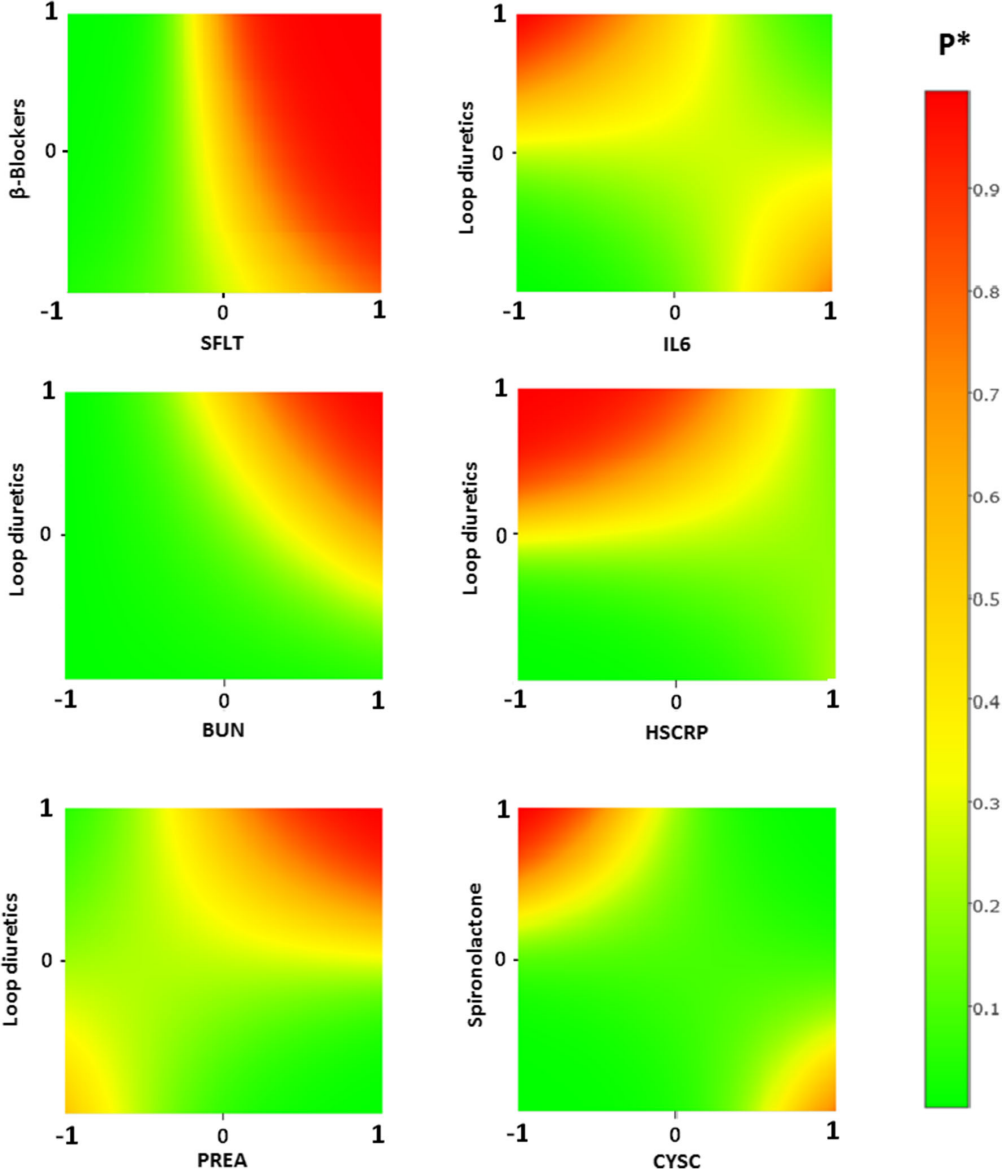
Effect of different levels of HF medications and biomarkers on the risk of HF hospitalization or death. *P: probability of HF hospitalization or death in a month. Range of biomarkers (in logarithmic form) and medications are standardized between − 1 and 1, for the range of the biomarker and medication concentration in our population

Figure 2 depicts the models with *P* value of biomarker—medication interaction lower than 0.05, for which RAMCD-CV > 0.7. It shows the effect of different levels of HF medications on probability of HF hospitalization or death in a month for patients with different levels of biomarkers. It suggests a beneficial effect of higher doses of loop diuretics in patients with high IL6 and/or high hsCRP. Higher loop diuretic doses seem to have adverse effects in patients with low hsCRP and/or high BUN and PREA. In contrast, high doses of loop diuretics do not seem to harm in patients with low BUN and PREA values. Spironolactone was associated with better outcome (low risk of HF or death) for patients with high CysC. It also demonstrates that up-titration of β-blockers for patients with high sFlt might not decrease the risk of HF or death, but that such patients might be better off with low β-blockers doses. Table 3 summarizes the main findings and provides potential therapeutic implications based on the results.

**Table 3:** Summary of hypothesis and main results of the study and potential future clinical implications

	This study	Potential future clinical impact
Hypothesis		
	Circulating biomarkers may predict response to single HF drugs in individual patients with HFrEF regarding outcome.	Tailored drug treatment in HFrEF patients, i.e., patients receive high doses of drugs only if they benefit from them, but low doses (or even no) if not required or potentially harmful.
Results		
sFlt levels	Low dose β-blocker beneficial if sFlt-levels were high	High sFlt ➔ no up-titration or reduction of β-blocker. Use other HF drugs first.
IL6/hs-CRP	High-dose loop diuretics beneficial if inflammation markers were high. Opposite if markers were low.	High inflammation markers ➔ increase loop diuretics.
		Low inflammation markers ➔ reduce loop diuretics.
BUN	High loop diuretics harmful if BUN was high.	Poor renal function ➔ increases spironolactone and reduced loop diuretics. Good renal function ➔ avoids high spironolactone doses and use loop diuretics liberally.
CysC	High spironolactone dose beneficial if CysC levels were high. Opposite of CysC levels were low.	
PREA	High doses of loop diuretics beneficial if PREA levels are low.	Low PREA levels ➔ increase loop diuretics.

## Discussion

This study investigated the hypothesis whether biomarkers may be able to predict the response to therapy in HF. Several significant biomarker treatment-effect interactions were found. We consider these results as promising signals which may predict a specific response to therapy in individual patients. It must be noted that this study is purely hypothesis generating regarding a novel approach to personalize medicine in HF with the use of biomarkers. Clearly, the results of this study need to be tested in prospective intervention studies before individualized therapy may be applied to HF patients. But our data provide the first attempt to not just predict outcome, but rather to select specific HF therapy based on individual biomarker patterns with the aim to improve outcome.

The need for such an individualized therapy approach in HF has been raised on many occasions, supported by several facts. Thus in real-life practice, there is still a very high mortality and morbidity despite drug development in the past decades [26]. Moreover, it is often difficult or sometimes even impossible to establish guideline-recommended drug therapy in HF [3, 4]. In such cases, it would be crucial to know which drug may be most important to be given in high doses to improve prognosis and which may be less important. At present, it is impossible to make this decision for an individual patient, and HF drugs have been investigated on top of previous established therapies only, i.e., β-blockers on top of ACE inhibitors and MRAs on top of both previous drugs. Despite a beneficial effect of these drugs as shown in large trials, not every drug will always have the same benefit in a specific patient or patient subgroup. Given the large heterogeneity of HF patients in terms of etiology and comorbidities, a “one-size-fits-all” approach is likely not optimal [26]. The dose of loop diuretics is even more so a clinical challenge and is largely intuitive. Guidelines recommend diuretics for symptom relief of congestion and recommend to lower diuretics whenever possible [3], but in many instances, they are inappropriately withheld or maintained because of fear of renal dysfunction or fear of decompensation, respectively.

### Considerations regarding statistics

This investigation is highly strengthened by the fact that both the covariates (i.e., biomarkers at the beginning of a given time interval, medication, and clinical covariates) and the outcome (i.e., HF hospitalization or death at the end of a given time interval) have been measured at multiple points in time. These multiple time points help to make the interactions we were investigating clinically relevant, by only looking into the treatment effect that occurred after the measurement of the biomarker in more than just one single time point. However, the difficulty with investigating repeated measurements lies within the fact that the outcomes for a single patient are correlated, because a patient with a hospitalization is prone to suffer from a re-hospitalization or die. Moreover, repeated measurements of biomarkers, medication, and other covariates are correlated as well. Therefore, the correlation between outcomes and variation of covariates in time needs to be taken into account for proper analysis.

One approach available for analyzing such data is using survival analysis methods, like time-dependent Cox regression models or recurrent analysis methods such as the Prentice, Williams, and Peterson model [27]. However, these methods take into account the values of time-dependent covariates only at the time of events (e.g., time of death or HF hospitalization), and the covariate values between events are disregarded, which was not acceptable to address the objective of this analysis. More proper alternative methods are longitudinal analysis methods [28] that not only take into account correlation in hospitalizations but also can involve more information about the variation of biomarkers and medications and their interactions over time [22].

Among longitudinal methods, the GEE models [18–20] have become a very popular regression model in medical studies [29–33]. The most attractive property of GEE models is that the resulting estimation of regression coefficients of those models is easy to interpret, especially for binary outcomes. Moreover, applying GEE models, scholars may hypothesize different structures of correlation between outcomes, but the resulting estimation of regression coefficients of GEE models is consistent and asymptotically normal, even when the correlation structure is imprecisely specified [18, 20]. Another advantage of GEE models for binary outcome (logistic-GEE models) is that with the use of RAMCD-CV, not only we can assess the adequacy of the model but also we can assess how the obtained results will generalize to a future data set [22].

Therefore in this study, we applied the weighted logistic-GEE model that applies a logistic regression model not only for the first, but also for repeated hospitalizations or death to test the interaction of biomarkers with the treatment effects of medications over time.

### Underlying pathways of biomarker drug interactions

First, we found a significant interaction between sFlt and β-blockers suggesting that patients with a high sFlt concentration may have a worse outcome with higher doses of β-blockers as compared to those on lower doses. Patients with low sFlt levels have a more favorable outcome overall, largely irrespective of β-blocker dose in our population. This raises the hypothesis that up-titration of β-blockers should be avoided in patients with high sFlt levels. sFlt is the soluble form of the endothelial- and macrophage-bound VEGF-receptor Flt-1. sFlt is formed after alternative splicing of Flt-1 RNA [34] and acts as a decoy receptor, thereby inhibiting VEGF and PLGF. This is assumed to result in anti-angiogenetic and anti-inflammatory effects [34]. sFlt concentrations are increased in HF [35, 36], myocardial infarction [37], preeclampsia, and coronary disease [34], and higher concentrations of sFlt are associated with adverse outcome in these disease entities [35–39]. Higher sFlt is also associated with more severe HF according to NYHA class and NT-proBNP [35, 38]. Nevertheless, the exact role of sFlt in the pathophysiology of HF and cardiovascular disease is not yet fully unraveled. sFlt-knockout mice developed more overt HF after aortic ligation, but on the other hand, administration of adenovirus expressing sFlt-1 caused diastolic dysfunction and decreased vascular density in wildtype mice [34]. Thus, both extremes of sFlt may have negative effects in the pathogenesis of HF, requiring a precise balance in the sFlt /PLGF pathway for adequate homeostasis. Another explanation could be that—analogous to natriuretic peptides—sFlt production is on itself a protective response to cardiac or vascular injury, but sFlt is associated with worse outcome because it also reflects the presence and magnitude of the injury itself. With regard to the interaction of sFlt with β-blockers on outcome in our study, it is interesting to note that baseline β-blocker use was independently associated with a lower baseline sFlt concentration previously [38]. Although this was a cross-sectional finding, it might suggest that β-blockers lower sFlt, but if this cannot be achieved, high doses of β-blockers might be less favorable. Another explanation could be that an elevated sFlt reflects an advanced stage of HF where β-blockers are difficult to up-titrate and might result in (temporary) deterioration. This would mean that other drugs might be given first to improve HF and reduce sFlt and β-blocker up-titration might be postponed. In addition, β-blockers have been found to have anti-angiogenetic effects in cancer [40]. Although to the best of our knowledge, such anti-angiogenetic effects of β-blockers have not been properly investigated in HF, it might be speculated that high levels of sFlt acting anti-angiogenetically may cause the potential anti-angiogenetic effects of β-blockers becoming evident. Obviously, this explanation is speculative and it needs to be investigated in animal studies if there is indeed such an interaction in HF.

Second, there was a significant interaction between CysC and spironolactone. Thus, patients with a low CysC had a less favorable prognosis on higher doses of spironolactone compared to those on lower doses, whereas patients with a high CysC had a better outcome with high versus low doses of spironolactone. We note that the results showed also the same pattern for BUN and creatinine, although the interactions were not significant (data is not shown). CysC is associated with inflammation and is the most sensitive marker of renal function in terms of glomerular filtration rate [41]. CysC is strongly associated with risk of cardiovascular disease (CVD) and adverse outcome in HF, but also in the general population. Despite some biological plausible links between CysC and CVD and HF, a recent Mendelian randomization study found no causative role for CysC in the development of CVD nor in the development of HF. Nevertheless, it remains a very reliable biomarker of high risk of events and disease progression in HF. We are not aware of any previous publication about the interaction between CysC and MRAs such as spironolactone. However, we and others previously found a similar treatment interaction with serum creatinine levels [16, 42]. A possible explanation could be that an impaired renal function reflected by elevated CysC or creatinine in the light of chronic HF is usually a form of cardiorenal syndrome which can be improved when HF is improved. Our results suggest that in this case, the preferred HF drug could be spironolactone. MRAs are considered to have anti-fibrotic and anti-inflammatory effects, which can also support the link between CysC and spironolactone that we found.

Finally, we found four biomarkers that interacted significantly with loop diuretic dose and outcome. First, in patients with high levels of BUN, higher doses of loop diuretics (HDLD) were associated with worse outcome, while this negative association with HDLD was not observed in patients with low BUN. Similar results were previously found by Testani et al. [43] evaluating 2456 patients in the BEST trial. In that study, HDLD was associated with worse outcome when BUN was ≥ 21 mg/dl, but this was not the case when BUN was low. In fact, after controlling for possible confounders, HDLD actually was associated with improved survival in those with low BUN, but with reduced survival in those with high BUN [43]. This interaction between BUN and loop diuretics was confirmed by Nunez et al. [44], who further elaborated on this by adding CA125 to the model, leading to a further specification of subgroups with differential risk associated with HDLD. Also supportive of our findings, higher levels of BUN were previously associated with poor diuretic response in HF patients with acute decompensation [45]. PREA revealed an interaction with loop diuretics in a similar direction—HDLD was associated with worse outcome when PREA was high, whereas HDLD was associated with good outcome when PREA was low. A recent study linked signs of intestinal congestion with elevated right atrial pressures and with cachexia [46]. Additionally, PREA was lower in patients with hypoalbuminemia [47], and both low PREA itself [48] and the presence of hypoalbuminemia [47] were associated with adverse outcome. This supports the idea that patients with low concentrations of PREA could have a benefit of HDLD because low PREA indirectly reflects a state of chronic venous congestion. Additionally, both low PREA [48] and cachexia in HF [46] are linked with increased inflammation, linking this interaction also to the inflammation markers. In this regard, two markers of inflammation—HSCRP and IL6—showed similar patterns of interaction with loop diuretics on outcome in the present analysis. Thus, when inflammation markers were low, HDLD was associated with an increased risk, whereas when inflammation markers were high, HDLD was associated with lower risk. Pro-inflammatory activation is linked to congestion [46, 49], but is also considered a major underlying mechanism of HF progression and a poor prognostic factor, supporting the interaction we found for inflammatory markers and loop diuretics.

### Limitations

This is a post hoc exploratory analysis of data from a randomized trial. Therefore, our results must be seen as means to identify potential relationships and to generate hypotheses. Further research—preferably prospective—is needed to confirm these interactions and their mechanisms. We are not aware of any other study in HF patients where repeated biomarker measurements and such detailed information on medication are available for retrospective validation of our results. In addition, animal studies are required to test the hypotheses raised by our findings. Possible limitations of this analysis are selection bias, reverse causality, and residual confounding factors. Nevertheless, because patients in the TIME-CHF trial were all attempted to be up-titrated on HF drugs either based on clinical symptoms and/or based on NT-proBNP levels, this may limit the selection bias for starting or up-titrating drugs compared to other cohorts. RAS inhibitors were given in rather high doses in almost all patients, and this may also explain why we did not find any interactions between RAS inhibition and biomarkers. Finally, stratification of patients into important subgroups would provide additional insight into the interaction between biomarkers and treatment response. However, the number of patients is not sufficient to reliably perform such analyses. Nevertheless, interactions between treatment response and comorbidities have been found in the main analysis [14]. Although different comorbidities were considered in multivariable analyses, not significantly influencing the results, the statistical model may not fully account for potential differences in subgroups.

### Conclusion

Our analysis suggests that repeated measurements of biomarkers might be helpful to individually tailor HF treatment to optimize the balance between beneficial and adverse effects of HF drugs. This might also be economically beneficial since patients would have better outcome (less hospitalizations, less side effects) with less medication, thereby reducing costs. However, this novel predictive, preventive, and personalized medicine approach clearly needs confirmation in other studies. Our data provide ground for prospective testing which will be needed before this novel and innovative concept can be adopted.

### Expert recommendations

Decision-making using such novel multiple biomarker approach may be very promising in bringing treatment of heart failure to a new level in the context of predictive, preventive, and personalized medicine. Table 3 provides potential clinical implications to adapt individual medication based on biomarker levels. Obviously, prospective testing for multiple drugs to guide individualized therapy should be different from standard randomized clinical trials, including sequential, multiple assessment, randomized trials (https://methodology.psu.edu/ra/adap-inter).

## Electronic supplementary material

ESM 1 (DOCX 72 kb)
