# Supplementary material for: Novel concept to guide systolic heart failure medication by repeated biomarker testing—results from TIME-CHF in context of predictive, preventive, and personalized medicine
Source: EPMA J. 2018 May 13;9(2):161–73. doi: 10.1007/s13167-018-0137-7 (PMC5972133; doi:10.1007/s13167-018-0137-7)
Supplement: Supplementary file 1 — (DOCX 72 kb) [file 13167_2018_137_MOESM1_ESM.docx]

**SUPPLEMENTAL MATERIAL**

Novel concept to guide systolic heart failure medication by repeated biomarker testing - TIME-CHF study

Nasser Davarzani^1,2,3,*^, M.Sc; Sandra Sanders-van Wijk^2^, MD, PhD; Micha T. Maeder^4^, MD; Peter Rickenbacher^5^, MD; Evgueni Smirnov^1^, PhD; Joël Karel^1^, PhD; Thomas Suter^6^, MD; Rudolf A. de Boer^7^, MD, PhD; Dirk Block^8^, PhD; Vinzent Rolny^8^, Dipl-Stat; Christian Zaugg^9^, PhD; Matthias E. Pfisterer^10^, MD; Ralf Peeters^1^, PhD; Hans-Peter Brunner-La Rocca^2,10^, MD; for the TIME-CHF investigators

(1) Maastricht University, Department of Data Science and Knowledge Engineering, Maastricht, the Netherlands

(2) Maastricht University Medical Center, Department of Cardiology, Maastricht, the Netherlands

(3) Maastricht University Medical Center, GROW School for Oncology and Developmental Biology, Department of Pathology, Maastricht, the Netherlands

(4) Kantonsspital St.Gallen, Department of Cardiology, St. Gallen, Switzerland

(5) University Hospital Bruderholz, Division of Cardiology, Bruderholz, Switzerland

(6) University Hospital Berne, Department of Cardiology, Berne, Switzerland

(7) University Medical Center Groningen, Department of Cardiology, Groningen, the Netherlands

(8) Roche Diagnostics GmbH, Penzberg, Germany

(9) Roche Diagnostics International, Rotkreuz, Switzerland

(10) University Hospital Basel, Department of Cardiology, Basel, Switzerland

*Address of correspondence: Maastricht University, Department of Data Science and Knowledge Engineering, St. Servaasklooster 39, P.O. Box 616, 6200 MD, Maastricht, the Netherlands. Tel: +31 (0)43 38 84803. Email: [n.davarzani@maastrichtuniversity.nl](mailto:n.davarzani@maastrichtuniversity.nl).

**Supplementary table 1: Overview of assays, measuring range and reference values.**

| **Biomarker** | **Assay** | **Type of test** | **Analyzer** | **Measuring range** | **Reference values** |
| --- | --- | --- | --- | --- | --- |
| **NT-proBNP** | proBNP II,  Roche diagnostics | Sandwich ELISA  immunoassay | Roche/Hitachi  cobas C systems | 5-35000 pg/mL | 300 pg/mL* |
| **hsTnT** | Troponin T hs,  Roche diagnostics | Sandwich ELISA  immunoassay | Roche/Hitachi  cobas C systems | 3-10000 pg/mL | 14 pg/mL |
| **GDF-15** | GDF15,  Roche diagnostics | ELISA | Microtiter plate  format,  not automated | 15.625-1000pg/mL,  LOD=22 pg/ml | 1532 pg/mL |
| **ST2** | PresageTM,  Critical Diagnostics | Sandwich ELISA  immunoassay | BEP® 2000  instrument  (Siemens) | LOB=0, LOD=1.31,  LOQ=2.35 ng/mL | 45.6 ng/ml |
| **tP1NP** | Total P1NP,  Roche diagnostics | Sandwich ELISA  immunoassay | Roche/Hitachi  cobas C systems | 5-1200 ng/mL | 76.31ng/ml |
| **hsCRP** | CRPHS,  Roche diagnostics | Particle-enhanced  immunoturbidi-  metric assay | Roche/Hitachi  cobas C systems | 0.1–20 mg/L;  LOD= 0.03 mg/L; | 5.0 mg/L |
| **IL6** | IL-6,  Roche diagnostics | Sandwich ELISA  immunoassay | Roche/Hitachi  cobas C systems | 1.5-5000 pg/mL | 7 pg/mL |
| **sFlt** | sFlt-1,  Roche diagnostics | Sandwich ELISA  immunoassay | Roche/Hitachi  cobas C systems | 10-85000 pg/mL;  LOQ =15, LOD=10,  LOB=6 pg/mL | 76.5 pg/mL ** |
| **PLGF** | PlGF,  Roche diagnostics | Sandwich ELISA  immunoassay | Roche/Hitachi  cobas C systems | 3-10000 pg/mL  LOB=2,  LOD=3,  LOQ=10 pg/mL | 3.45 pg/mL ** |
| **PREA** | PREA,  Roche diagnostics | Immunoturb-  dimetric assay | Roche/Hitachi  cobas C systems | 0.03-0.8 g/L | 0.2 g/L |
| **Uric acid** | UA2,  Roche diagnostics | Enzymatic colori-  metric test | Roche/Hitachi  cobas C systems | 0.2-25.0 mg/dL | Males: 3.4-7.0 mg/dL  Females: 2.4-5.7 mg/dL |
| **Creatinine** | Creatinine,  Roche diagnostics | Kinetic colori-  metric assay | Roche/Hitachi  cobas C systems | 18–2210 μmol/l | Males: 62–106 μmol/l  Females: 44–80 μmol/l |
| **BUN** | UREAL,  Roche diagnostics | Kinetic photo-  metric asasy | Roche/Hitachi  cobas C systems | 0.5-40 mmol/L | 2.76-8.07 mmol/L |
| **CysC** | Cystatin C,  Roche diagnostics | Particle enhanced  immunoturbidi-  metric assay | Roche/Hitachi  cobas C systems | 0.4–8.0 mg/L | - 1. g/L |
| **Ferritin** | Ferritin, Roche diagnostics | Particle enhanced  immunoturbidi-  metric assay | Roche/Hitachi  cobas C systems | 5-1000 ng/mL | Males: 30-400 μg/L  Females: 15-150 μg/L |
| **SHBG** | SHBG, Roche diagnostics | Sandwich ELISA  immunoassay | Roche/Hitachi  cobas C systems | 0.800‑200 nmol/L | Males  ≥ 50 yrs: 40 nmol/L  Females  ≥ 50 yrs: 57 nmol/L |
| **sTFR** | STFR, Roche diagnostics | Sandwich ELISA  immunoassay | Roche/Hitachi  cobas C systems | 0.50‑40.0 mg/L (5.9‑472 nmol/L, 0.05‑4.00 mg/dL) | Males:  2.2‑5.0 mg/L 26‑59 nmol/L 0.22‑0.50 mg/dL  Females:  1.9‑4.4 mg/L 22‑52 nmol/L 0.19‑0.44 mg/dL |
| **OPN** | OPN, Roche diagnostics | Sandwich ELISA  immunoassay | Roche/ Microtiterplate | R&D assay | R&D assay |
| **Mimican** | Mimican, Roche diagnostics | Sandwich ELISA  immunoassay | Roche/ Microtiterplate | R&D assay | R&D assay |
| **IGFBP7** | IGFBP7, Roche diagnostics | Sandwich ELISA  immunoassay | Roche/ Microtiterplate | R&D assay | R&D assay |
| LOD=limit of detection; LOB=limit of blank, LOQ=limit of quantification, ULN=upper limit of normal. Data is substracted from package inserts from the manufacturer, unless otherwise indicated.  *Rule-out value for heart failure.  **Border of outliers of our own dataset was used as reference value as no representative reference values are available – i.e. only in pregnant woman. | | | | | |

| **Supplementary table 2: Layout of dataset for applying logistic-GEE models.** | | | | | |
| --- | --- | --- | --- | --- | --- |
| Patient ID | HF Hospitalization or death in the next month | Time point | Medication | Biomarker and time-dependent covariates | Fixed covariates |
| 1 | 0 | M_0_ | M_0_ | M_0_ | M_0_ |
| 1 | 1 | M_1_ | Avg(M_0_, M_1_) | M_1_ | M_0_ |
| 1 | 0 | M_2_ | Avg(M_1_, M_2_) | M_1_ | M_0_ |
| 1 | 0 | M_3_ | Avg(M_2_, M_3_) | M_3_ | M_0_ |
| 1 | 0 | M_4_ | Avg(M_3_, M_4_) | M_3_ | M_0_ |
| 1 | 1 | M_5_ | Avg(M_4_, M_5_) | M_3_ | M_0_ |
| 1 | 1 | M_6_ | Avg(M_5_, M_6_) | M_6_ | M_0_ |
| 2 | 0 | M_0_ | M_0_ | M_0_ | M_0_ |
| 2 | 0 | M_1_ | Avg(M_0_, M_1_) | M_1_ | M_0_ |
| 2 | 1 | M_2_ | Avg(M_1_, M_2_) | M_1_ | M_0_ |
| 2 | 1 | M_3_ | Avg(M_2_, M_3_) | M_3_ | M_0_ |
| 2 | 1 | M_4_ | Avg(M_3_, M_4_) | M_3_ | M_0_ |
| **.**  **.**  **.** | .  .  . | .  .  . | .  .  . | .  .  . | .  .  .  .  .  ..  . |
| 499 | 0 | M_0_ | M_0_ | M_0_ | M_0_ |
| 499 | 0 | M_1_ | Avg(M_0_, M_1_) | M_1_ | M_0_ |
| 499 | 0 | M_2_ | Avg(M_1_, M_2_) | M_1_ | M_0_ |
| 499 | 1 | M_3_ | Avg(M_2_, M_3_) | M_3_ | M_0_ |
| 499 | 1 | M_4_ | Avg(M_3_, M_4_) | M_3_ | M_0_ |
| 499 | 1 | M_5_ | Avg(M_4_, M_5_) | M_3_ | M_0_ |
| 499 | 0 | M_6_ | Avg(M_5_, M_6_) | M_6_ | M_0_ |
| 499 | 0 | M_7_ | Avg(M_6_, M_7_) | M_6_ | M_0_ |
| 499 | 1 | M_8_ | Avg(M_7_, M_8_) | M_6_ | M_0_ |
| 499 | 0 | M_9_ | Avg(M_8_, M_9_) | M_6_ | M_0_ |
| 499 | 0 | M_10_ | Avg(M_9_, M_10_) | M_6_ | M_0_ |
| 499 | 1 | M_11_ | Avg(M_10_, M_11_) | M_6_ | M_0_ |
| 499 | 0 | M_12_ | Avg(M_11_, M_12_) | M_12_ | M_0_ |
| 499 | 0 | M_13_ | Avg(M_12_, M_13_) | M_12_ | M_0_ |
| 499 | 1 | M_14_ | Avg(M_13_, M_14_) | M_12_ | M_0_ |
| 499 | 0 | M_15_ | Avg(M_14_, M_15_) | M_12_ | M_0_ |
| 499 | 1 | M_16_ | Avg(M_15_, M_16_) | M_12_ | M_0_ |
| 499 | 0 | M_17_ | Avg(M_16_, M_17_) | M_12_ | M_0_ |
| 499 | 0 | M_18_ | Avg(M_17_, M_18_) | M_18_ | M_0_ |
| M_i_: month i; M_0_: baseline; Avg: average | | | | | |


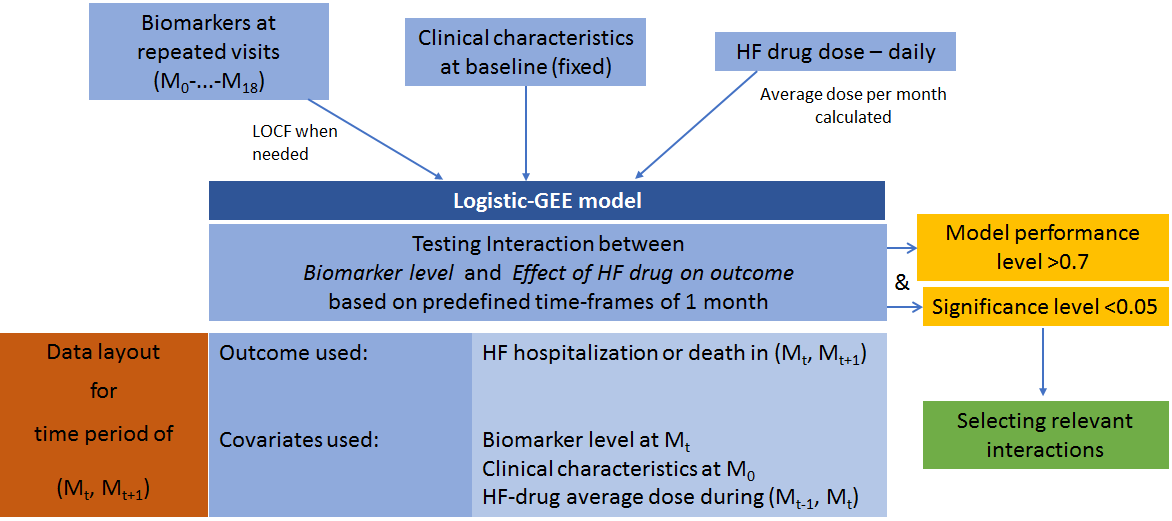


**Supplementary Figure 1. Method layout for assessing the interactions between biomarkers and medications.** M_i_: month i; M_0_: baseline. LOCF: last observation carried forward method.

**1.** Preisser JS, Lohman KK, Rathouz PJ. Performance of weighted estimating equations for longitudinal binary data with drop-outs missing at random. Stat Med 2002;21:3035-3054.
